# A transgenerational toxicokinetic model and its use in derivation of Minnesota PFOA water guidance

**DOI:** 10.1038/s41370-018-0110-5

**Published:** 2019-01-10

**Authors:** Helen M. Goeden, Christopher W. Greene, James A. Jacobus

**Affiliations:** Minnesota Department of Health, 625 Robert St. N, P.O. Box 64975, St. Paul, MN 55164-0975 USA

## Abstract

Minnesota has been grappling with extensive per- and polyfluoroalkyl substances (PFASs) groundwater contamination since 2002, in a major metropolitan setting. As toxicological information has accumulated for these substances, the public health community has become increasingly aware of critically sensitive populations. The accumulation of some PFAS in women of childbearing age, and the placental and breastmilk transfer to their offspring, require new risk assessment methods to protect public health. The traditional water guidance paradigm is inadequate to address maternal-to-infant transfer of accumulated levels of perfluorooctanoate (PFOA), in particular. Even short exposures during infancy have dramatic impacts on serum levels for many years. In addition, developmental effects are the critical effects anchoring recent risk assessments. In response, the Minnesota Department of Health created an Excel-based model that incorporates chemical-specific properties and exposure parameters for early life stages. Serum levels were assessed in both formula-fed and breastfed infants, with placental transfer in both scenarios. Peak breastfed infant serum levels were 4.4-fold higher than in formula-fed infants, with both of these scenarios producing serum levels in excess of the adult steady-state level. The development and application of this model to PFOA are described.

## Introduction

Per- and polyfluoroalkyl substances (PFASs) are a group of fluorinated organic pollutants with over 60 years of widespread industrial and commercial use. These water contaminants are highly problematic due to their water solubility, high persistence, and bioaccumulation, especially in humans. The increasing detection of these contaminants, as well as increasing concerns regarding potential adverse health effects, have resulted in their emergence as drinking water contaminants of global concern.

**Fig. 1:**
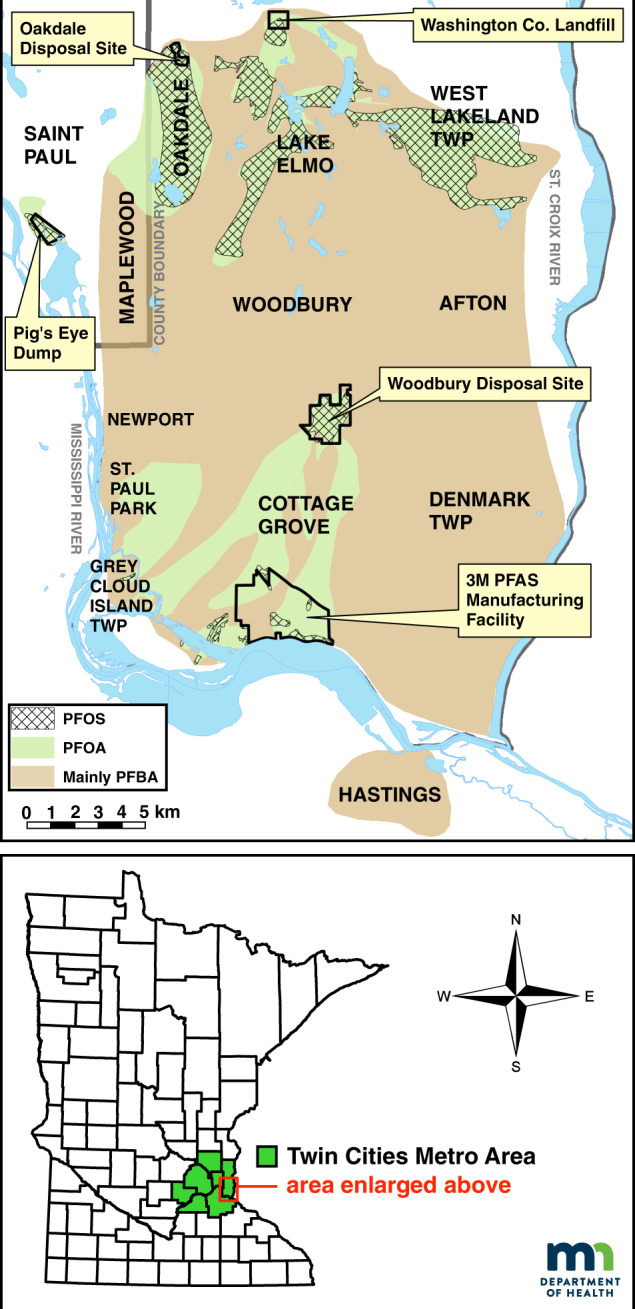
Map of PFAS-impacted area east of St. Paul, Minnesota metropolitan area

In Minnesota, since 2002, the Minnesota Department of Health (MDH), in partnership with the Minnesota Pollution Control Agency (MPCA), has been involved in investigating PFAS contamination. This work began when MDH received a request to develop health-based guidance values (HBGVs) for two PFAS chemicals, perfluorooctane sulfonate (PFOS) and perfluorooctanoate (PFOA), to assist in evaluating human health risks associated with groundwater contamination at the 3M Corporation’s Cottage Grove manufacturing plant (see Fig. 1).

In 2004, PFOS and PFOA contamination was detected in the drinking water supplies of several eastern Twin Cities suburbs (East Metro). These contaminants originate from three sites used by the 3M Corporation over several decades for disposal of PFAS manufacturing wastes. In response, MDH and MPCA began extensive testing of public and private wells in the area for PFOS and PFOA. In 2006, the MDH Public Health Laboratory developed new analytical methods, expanding the list of chemicals to include five more PFAS: perfluorobutanoic acid (PFBA), perfluoropentanoic acid (PFPeA), perfluorohexanoic acid (PFHxA), perfluorobutane sulfonate (PFBS), and perfluorohexane sulfonate (PFHxS). To date, multiple public water supplies and over 2600 private wells have been sampled. The East Metro PFAS groundwater contamination plume currently covers over 150 square miles, affecting the drinking water supplies of over 140,000 Minnesotans. PFBA is the most widely detected PFAS, whereas PFOA, PFOS, and other PFAS are present over a smaller area (Fig. 1). Statewide, MDH and MPCA have evaluated other potential sources of PFAS contamination, including fire-training facilities, chrome plating operations, wastewater treatment plants, and landfills. Low concentrations of PFAS were detected at many of these locations, often below the threshold of human health concern, although these thresholds continue to decline as more information becomes available.

MDH derives HBGVs to assist risk managers in identifying water sources with contaminants at levels of potential human health concern. An HBGV represents a concentration in drinking water of a chemical or mixture of chemicals that is likely to pose little or no health risk to humans, including vulnerable subpopulations. To protect the majority of the general population, MDH uses a reasonable maximum exposed (RME) individual scenario, which uses central tendency values for some parameters coupled with upper-end values for others (e.g., 95th percentile water intake rate) [1]. Following the 2016 issuance of lifetime health advisories (HAs) of 0.07 µg/L for PFOS and PFOA by the US Environmental Protection Agency (USEPA) [2, 3], MDH initiated an expedited reassessment of Minnesota’s PFOS and PFOA HBGVs.

In its reassessment, MDH found that its standard approach for deriving HBGVs was inadequate when applied to PFOS and PFOA for several reasons. PFOS and PFOA are bioaccumulative chemicals, resulting in higher serum concentrations than the concentrations in environmental media (e.g., water). Recent studies have demonstrated significant maternal transfer across the placental barrier, resulting in measurable neonatal serum concentrations at birth [4–7], and partitioning into breastmilk [7–10]. Empirical data from these populations clearly demonstrate higher serum levels of PFOS and PFOA in nursing infants compared with their mother. Kinetic models of infant serum levels also predict several fold higher serum levels following breastfeeding [11, 12]. Therefore, in addition to being born with a transgenerational body burden from placental transfer based on maternal accumulation, infants may also experience subsequent higher exposures, especially from breastfeeding. Developmental effects have been identified as sensitive health effects; therefore, consideration of these exposure pathways is relevant and likely even critical to protection of all sensitive subpopulations. For these reasons, MDH developed a new approach to derive HBGVs, accounting for bioaccumulation and transgenerational exposure.

This publication presents the development and application of a flexible and transparent Excel-based toxicokinetic (TK) model, as applied to water guidance derivation for PFOA. The model incorporates body burden at birth (placental transfer), ingestion of breastmilk, and age-specific water intake rates in order to derive sufficiently protective HBGVs.

## Materials and methods

### MDH’s TK maternal/infant model approach for deriving HBGVs

**Fig. 2:**
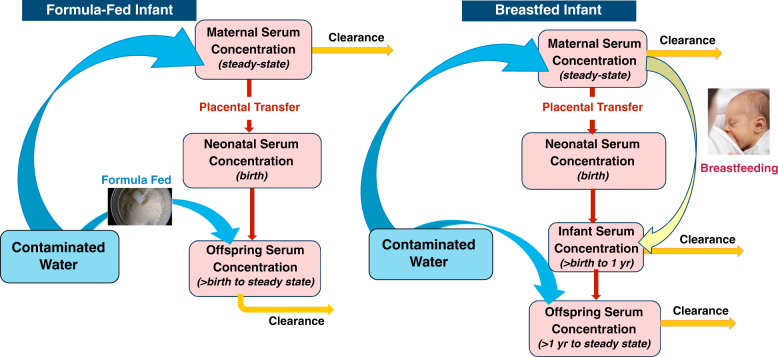
Conceptual representation of the toxicokinetic model for the two exposure scenarios evaluated

MDH developed an Excel-based TK model to predict serum levels from birth through adulthood. MDH chose to develop its model in Excel to maximize the transparency and accessibility of the model. In addition, the relationship between intake (dose) and serum concentration can adequately be described by a single-compartment model [13]. This type of model has been used by others to describe the relationship between dose and serum levels [14]. Two exposure scenarios were evaluated (Fig. 2): (1) an infant fed with formula reconstituted with contaminated water starting at birth, followed by a lifetime of drinking contaminated water; and (2) an infant breastfed for 12 months, followed by a lifetime of drinking contaminated water. In both scenarios, infants began life with a transgenerational body burden calculated from the mother’s serum concentration using a placental transfer factor. Exposure was simulated through consumption of breastmilk or formula reconstituted with contaminated water. Daily intake, elimination, and serum concentrations were calculated over a simulated period of 20,000 days (about 55 years) to ensure attainment of steady state (See Table 1).

**Table 1:** Exposure and chemical-specific toxicokinetic parameters used in modeling PFOA serum concentrations

Parameter	Central tendency value (e.g. mean)	Upper percentile value (e.g., 95th percentile)	Source and comment	
Half-life (t_½_)	**840 days [2.3 years]**	1679 days [4.6 years]	Bartell et al. [37]. Similar mean values have been reported in several publications. Wide variability within the general population has been noted. Life-stage-specific information is not available. Therefore, the same half-life was used across all life stages. See Supplemental Table S1 for more information.	
Placental (infant:maternal) transfer	**0.87**	1.69	Mean paired ratios ranging from 0.68 [38] to 1.26 [4] have been reported. A more comprehensive literature review has been conducted since MDH selected 0.87 during its expedited review [16]. See Supplemental Table S2. Preference was given to paired ratios over ratios based on summary statistics. Maximum ratios for individual mother–infant pairs range from 1.52 [7] to 2.16 [6]. 95th percentile values (mean + 2SD) were calculated from author-reported mean and SD values, and range from 1.11 [7] to 1.69 [4].	
Breastmilk (milk:maternal) transfer	**0.052**	0.12	Mean paired ratios ranging from 0.025 [39] to 0.12 [40] have been reported. A more comprehensive literature review has been conducted since MDH selected 0.052 during its expedited review [16]. See Supplemental Table S3. Maximum individual pair ratios were not reported by study authors. In the absence of maximum individual values, the maximum mean value of 0.12 is used to represent an upper percentile value.	
Breastmilk intake rate (mL/kg per day)			Values for exclusively^a^ breastfed infants (Table 15-1 [36]). Body weight at birth was set at **3.38** kg, the mean birth weight for singleton births at 37–41 weeks of gestation [41]. Body weights (kg) were calculated from data presented in Table 15-1 for each age group (i.e., mL/day ÷ mL/kg per day): The midpoint in time for each age group was set equal to age group value. Daily intake rates and body weights between one midpoint and the next were calculated by linear interpolation to avoid abrupt changes in values.	
Age group			Mean based (kg)	Upper percentile-based (kg)
Birth to <1 month	150	**220**	3.4	**4.3**
1 to <3 months	140	**190**	4.9	**5.2**
3 to <6 months	110	**150**	7.0	**6.7**
6 to <12 months	83	**130**	7.5	**7.7**
Duration (months) of breastfeeding	6 months, then phased out to zero by 12 months	**12 months**	American Academy of Pediatrics [42] recommends exclusively breastfeeding for the first 6 months, with continued breastfeeding alongside introduction of complementary food for at least 12 months. The Center for Disease Control (CDC) Breastfeeding Report Card for 2016 [43] reports nearly 66% of mothers in Minnesota report breastfeeding at 6 months, with 31.4% exclusively breastfeeding. At 12 months, 41% of mothers reported breastfeeding. Central tendency: exclusively^a^ breastfed intake rates used from birth to 6 months of age. From 6 to 12 months, breastfeeding is phased out and water intake is phased in. **Upper percentile: exclusively breastfed intake rates used from birth to 12 months of age. At 12 months, breastfeeding ends and water intake begins**.	
Water intake rate (mL/kg per day)			Values for consumers only. (Table 3-1 [36]). Body weights (kg) were calculated from data presented in Table 3-1 for each age group (i.e., mL/day ÷ mL/kg per day): The midpoint in time for each age group was set equal to age group value. Daily intake rates and body weights between one midpoint and the next were calculated by linear interpolation to avoid abrupt changes in values. **For calculation of maternal serum concentration at time of delivery, a time-weighted average water intake rate was calculated from birth to 30–35 years of age**, resulting in a mean and **95th percentile water intake rate** of 18 and **47** **mL/kg per day**, respectively.	
Age group			Mean based (kg)	Upper percentile based (kg)
<1 month	137	**238**	3.4	**3.6**
1 to <3 months	119	**285**	4.6	**3.7**
3 to <6 months	80	**173**	7.0	**6.8**
6 to <12 months	53	**129**	8.8	**8.9**
1 to <2 years	27	**75**	11. 4	**11.9**
2 to <3 years	26	**62**	13. 7	**14.7**
3 to <6 years	21	**52**	18. 2	**19.2**
6 to <11 years	17	**47**	30. 1	**29.9**
11 to <16 years	12	**35**	53. 1	**56.5**
16 to <18 years	10	**30**	70. 2	**62.8**
18 to <21 years	11	**36**	74. 2	**78.3**
>21 years	16	**42**	76.7	**73.6**
Volume of distribution (L/kg)	**0.17**		USEPA and Han et al. [14, 44]. Consistent with extracellular fluid as volume of distribution.	
*V*_d_ age adjustment factor (*V*_d_ AF)			Friis-Hansen [15] (and consistent with Felter et al. [45]). Early life stages have higher body water content per unit weight than adults. Adjustment factor is designed to account for this difference. This is an area of uncertainty since the precise nature of the *V*_d_ is not known. Use of the *V*_d_ AF reduces serum concentration estimates, and increases model accuracy compared with empirical data. The midpoint in time for each age group was set equal to age group value. Daily *V*_d_ AF between one midpoint and the next were calculated by linear interpolation to avoid abrupt changes in values.	
0–1 day	**2.4**			
1–30 days	**2.1**			
1–3 months	**1.7**			
3–6 months	**1.6**			
6–12 months	**1.5**			
1–3 years	**1.4**			
3–5 years	**1.1**			
5–10 years	**1.2**			
>10 years	**1.0**			

Final model parameters for calculation of the PFOA HBGVs shown in **bold**

^a^Exclusively breastfed as defined by USEPA [36] refers to infants whose sole source of milk is breastmilk and not formula. Exclusively breastfed infants in the studies underlying these USEPA estimates were not excluded from other foods, typically after six months. This definition differs from other sources, which may define exclusive breastfeeding as the only source of nourishment (solid or liquid) as breastmilk

Because PFOA is well absorbed and not metabolized, the dynamic relationship between serum concentrations and intake (dose) can be calculated using Eq. 1:

1 $${{\mathrm{Serum}}\,{\mathrm{concentration}}\left( {\frac{{{\mathrm{mg}}}}{{\mathrm{L}}}} \right) = \frac{{{\mathrm{Dose}}\left( {\frac{{{\mathrm{mg}}}}{{{\mathrm{kg}} \cdot {\mathrm{day}}}}} \right)}}{{{\mathrm{Clearance}}\,{\mathrm{rate}}\left( {\frac{{\mathrm{L}}}{{{\mathrm{kg}} \cdot {\mathrm{day}}}}} \right)}}}$$

Where:

for water ingestion—

$${{\mathrm{Dose}}\left( {\frac{{{\mathrm{mg}}}}{{{\mathrm{kg}} \cdot {\mathrm{day}}}}} \right) {\mathrm{= Water}}\ {\mathrm{intake}}\,{\mathrm{rate}}\left( {\frac{{\mathrm{L}}}{{{\mathrm{kg}} \cdot {\mathrm{day}}}}} \right) {\mathrm{\times Water}}\,{\mathrm{concentration}}\left( {\frac{{{\mathrm{mg}}}}{{\mathrm{L}}}} \right)}$$

for breastmilk—

$${{\mathrm{Dose}}\left( {\frac{{{\mathrm{mg}}}}{{{\mathrm{kg}} \cdot {\mathrm{day}}}}} \right){\mathrm{ = Breastmilk}}\,{\mathrm{intake}}\,{\mathrm{rate}}\left( {\frac{\mathrm{L}}{{{\mathrm{kg}} \cdot {\mathrm{day}}}}} \right){\mathrm{ \times Breastmilk}}\,{\mathrm{concentration}}\left( {\frac{{{\mathrm{mg}}}}{\mathrm{L}}} \right)}$$

and

$${\mathrm{Clearance}}\,{\mathrm{rate}}\left( {\frac{\mathrm{L}}{{{\mathrm{kg}} \cdot {\mathrm{day}}}}} \right) = V_{\mathrm{d}} \times k$$

$$V_{\mathrm{d}} = {\mathrm{Volume}}\,{\mathrm{of}}\,{\mathrm{distribution}}\left( {\frac{\mathrm{L}}{{{\mathrm{kg}}}}} \right)$$

$$\hskip 10pt k = \frac{{{\mathrm{ln}}\left( 2 \right)}}{{{\mathrm{half - life}}\,{\mathrm{(d)}}}}$$

An annotated list of model exposure and chemical parameter values is presented in Table 1.

The model assumes that maternal exposure began prior to pregnancy, so that steady-state serum concentration was achieved by the time of delivery. The infant’s serum concentration at birth was calculated using Eq. 2:

2 $${{\mathrm{Serum}}\,{\mathrm{conc}}{\mathrm{.}}\left( {\frac{{{\mathrm{mg}}}}{\mathrm{L}}} \right){\mathrm{= Maternal}}\,{\mathrm{serum}}\,{\mathrm{conc}}{\mathrm{.}}\left( {\frac{{{\mathrm{mg}}}}{\mathrm{L}}} \right)}\\ \times {{\mathrm{Placental}}\,{\mathrm{transfer}}\,{\mathrm{factor}}}$$

For all subsequent days, the infant’s final daily post-elimination serum concentration was calculated using Eq. 3:

3 $$	{\mathrm{Serum}}\,{\mathrm{conc}}{\mathrm{.}}\left( {\frac{{{\mathrm{mg}}}}{{\mathrm{L}}}} \right) =\\ 	 \left[ {{\mathrm{Prev}}{\mathrm{.day}}\,{\mathrm{serum}}\,{\mathrm{conc}}{\mathrm{.}}\left( {\frac{{{\mathrm{mg}}}}{{\mathrm{L}}}} \right) \!+\! \frac{{{\mathrm{Today}}\prime {\mathrm{s}}\,{\mathrm{intake}}\left( {{\mathrm{mg}}} \right)}}{{V_{\mathrm{d}}\left( {\frac{{\mathrm{L}}}{{{\mathrm{kg}}}}} \right){\mathrm{ \times BW}}\left( {{\mathrm{kg}}} \right)}}} \right] \times e^{ - k}$$

The *V*_d_ parameter, assumed to be extracellular water, is both chemical specific and age specific. In order to account for age-specific differences in extracellular water volume during early childhood, *V*_d_ was multiplied by an adjustment factor (AF) starting at 2.1 at birth and declining to 1.0 by 10 years of age [15].

To maintain mass balance, daily maternal serum concentrations incorporated loss of chemical via transfer to the infant during breastfeeding, as well as excretion represented by the clearance rate. The infant’s daily intake (and thus the mother’s loss) was calculated from the breastmilk intake rate and the breastmilk concentration, derived using Eq. 4:

4 $${\mathrm{Breastmilk}}\,{\mathrm{conc}}{\mathrm{.}}\left( {\frac{{{\mathrm{mg}}}}{{\mathrm{L}}}} \right) = \ {\mathrm{Maternal}}\,{\mathrm{serum}}\,{\mathrm{conc}}{\mathrm{.}}\left( {\frac{{{\mathrm{mg}}}}{{\mathrm{L}}}} \right)\\ {\mathrm{ \times Breastmilk}}\,{\mathrm{transfer}}\,{\mathrm{factor}}$$

### Model evaluation

Model results were compared with empirical data from published studies to ensure that the model was fit-for-purpose, i.e., capable of generating representative upper percentile serum concentration estimates over a lifetime for a population of concern, in particular, infants breastfed by chronically exposed mothers. MDH also solicited input from six external experts for advice on how to improve the model predictions and for feedback regarding the suitability of the model for the intended purpose [16].

### Reference dose (RfD) calculation and relative source contribution (RSC) selection

Derivation of HBGVs typically requires determination of an RfD (mg/kg per day) and an appropriate RSC. However, serum concentration, a measure of internal exposure, was identified as the best dose metric for assessing PFOA’s health effects. The point of departure was a serum concentration of 38 mg/L from a developmental study in mice [17]. The application of a total uncertainty factor of 300 (10^0.5^ for potential interspecies toxicodynamic differences, 10 for intraspecies variability, 10^0.5^ for use of a lowest observable adverse effect level (LOAEL), and 10^0.5^ for database insufficiencies) produced a ‘reference’ serum concentration of 0.13 mg/mL. A traditional RfD of 0.000018 mg/kg per day can be derived by multiplying the ‘reference’ serum concentration of 0.13 mg/L by a clearance rate of 0.00014 L/kg per day [18].

Total exposure from all sources, including potential ingestion of contaminated drinking water, should not result in higher serum concentrations than those associated with the RfD (hereto referred to as ‘reference’ serum concentration). Exposures contributed by non-water sources are addressed through the application of an RSC, which allocates a fraction of the RfD to drinking water exposure. National and local biomonitoring data were used to identify an appropriate RSC for PFOA (see details in Results section).

## Results

### Comparison of breastmilk versus formula-fed exposure pathways

**Fig. 3:**
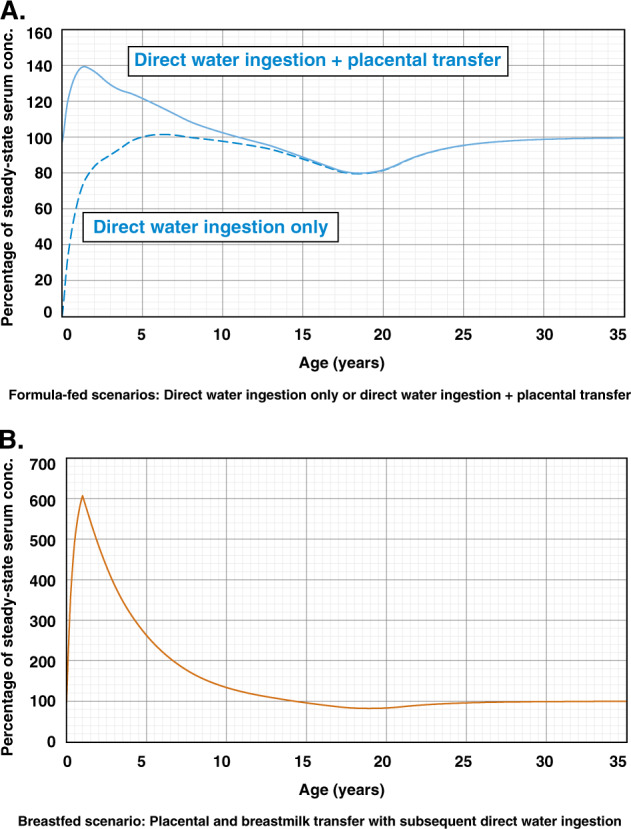
Offspring serum concentration as a percentage of steady-state serum concentration (**a**) formula-fed scenarios—direct water ingestion exposure only or placental transfer (from chronically exposed mother at steady state) and direct water ingestion exposure, and (**b**) breastfed scenario—placental and breastmilk transfer (from chronically exposed mother at steady state) and direct water ingestion exposure. Note different scales are used for percentage of steady-state concentration in **a** (0–160) than in **b** (0–700). (Horizontal scale truncated at 35 years to enhance detail)

MDH developed a preliminary model to evaluate whether placental and breastmilk transfer, as well as high fluid intake rates could result in serum concentrations that exceeded steady-state serum concentrations. Two formula-fed scenarios and one breastfed scenario were evaluated: a formula-fed infant exposed to contaminated water with or without placental transfer (Fig. 3a) and a breastfed infant with both placental and breastmilk transfer (Fig. 3b). Figure 3a demonstrates the importance of considering placental transfer, as early life serum levels are predicted to be approximately 40% higher than adult steady-state levels. When both placental and breastmilk transfer are taken into account (Fig. 3b), early life serum levels were predicted to be sixfold higher than adult steady-state levels. Given the impact of exposure via placental and breastmilk transfer, MDH pursued the development of a model that incorporated these pathways into the derivation of an HBGV for PFOA.

### Model evaluation

Empirical infant serum data [8, 19] were used to ascertain whether the Excel-based model produces reasonable estimates of serum concentration, keeping in mind that the model parameter selections assume an RME scenario. For each model comparison, the mother’s serum concentration at delivery was assumed to be at steady state. Individual maternal:child paired numeric data were preferred, but was not included in the publications or available by request.

**Fig. 4:**
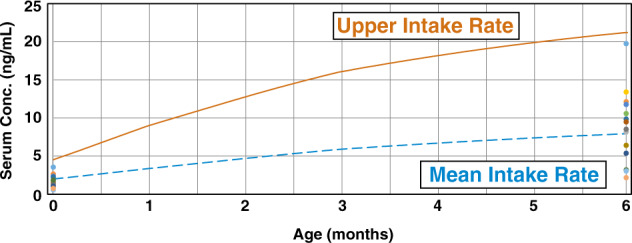
Mean and 95th percentile infant PFOA serum concentrations predicted by MDH’s model for breastfed infants in comparison with measured infant PFOA serum levels presented in Fromme (Table 1[8]). Upper and mean intake rates derived from USEPA [36] (see Table 1)

Fromme and colleagues [8] investigated maternal and infant PFOA body burden during the 6 months following birth. Breastfeeding status was reported for 50 of the 53 participants; 37 infants drank only breastmilk, 6 predominantly drank breastmilk, 6 partially drank breastmilk, and 1 infant received no breastmilk. Two comparisons were conducted: (1) a population-based evaluation, and (2) modeling of individual infant serum levels after 6 months of breastfeeding. For the population-based evaluation, the overall maternal mean (2.3 µg/L) and 95th percentile (5.2 µg/L) PFOA serum concentrations at delivery (Table 1 in Fromme et al. [8]) was input into the model. Maternal exposure during lactation was assumed to be the same as prior to delivery and was estimated by multiplying the maternal serum concentration by a PFOA clearance rate of 0.00014 L/kg per day, which is based on a 0.17 L/kg volume of distribution and a half-life of 840 days. Placental and breastmilk transfer rates of 0.87 and 0.052, respectively, were used to estimate infant serum concentrations at birth and breastmilk concentration from maternal serum concentrations over the course of lactation. Predicted serum concentrations, following 6 months of breastfeeding, aligned closely with the reported mean and 95th percentile infant serum concentrations at 6 months of age (Fromme Table 1 [8]). The reported overall mean and 95th percentile infant PFOA serum concentrations at 6 months were 8.0 and 19.5 µg/L, respectively, and the predicted values were 7.9 and 21.2 µg/L, respectively, based on mean (dashed line) and upper percentile (solid line) breastmilk intake rates (Fig. 4).

**Fig. 5:**
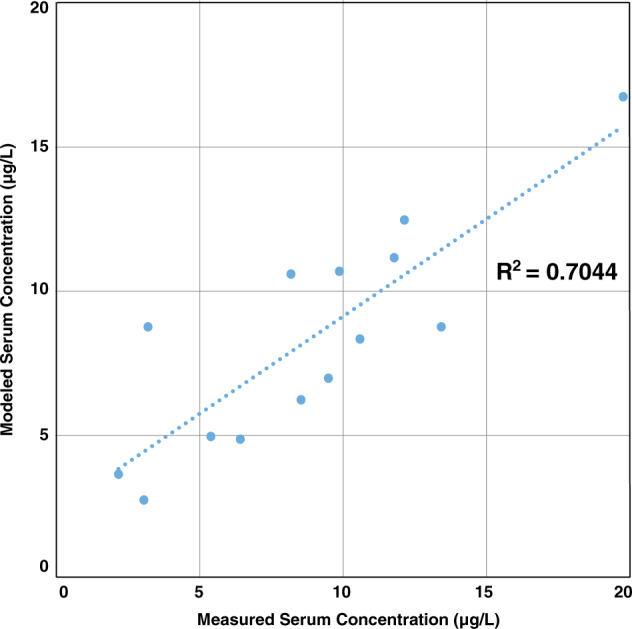
Modeled individual infant PFOA serum concentration at 6 months of age versus measured levels estimated from Fromme et al. (Figure S6 [8])

For modeling of individual serum concentrations, WebPlotDigitizer (Austin, Texas, USA) [20] was used to approximate the serum concentration at birth (cord blood) and at 6 months of age from Figure S6 [8] for each of the 14 infants and compared these values with the MDH model results. The reported birth serum concentration was used as the input to the model for each infant. An upper percentile breastmilk intake rate was used for the entire 6-month period. Maternal serum concentration at delivery was back calculated using the infant birth serum concentration and a placental transfer rate of 0.87. Initial breastmilk concentration was calculated using the estimated maternal serum concentration at delivery and a breastmilk transfer factor of 0.052. Total maternal exposure during lactation was assumed to be the same as prior to delivery and was calculated  by multiplying the maternal serum concentration by a clearance rate of 0.00014 L/kg per day. Model performance was evaluated using the coefficient of determination (*R*^2^) from linear regression of predicted versus measured infant serum levels. A comparison of predicted to the estimated measured infant serum concentrations at 6 months of age produced an *R*^2^ of 0.7044 (Fig. 5). On average, model predictions slightly (<10%) overestimated PFOA levels.

Mogensen and colleagues estimated or measured serum concentrations of PFOA in a Faroese birth cohort at delivery and 11, 18, and 60 months of age to determine the impact of breastfeeding [19]. This set of data is less optimal than Fromme for evaluating model performance for a variety of reasons, including the time interval between cessation of breastfeeding and serum sampling (see Supplemental Information). WebPlotDigitizer allowed estimation of serum concentrations for PFOA at birth and at 11 months of age from curves for 11 children, who were at least partially breastfed (as presented in Mogensen’s Fig. 1 [19]). Two comparisons were conducted: (1) magnitude of relative change in infant serum concentrations from birth to 11 months of age and (2) modeling of individual infant serum concentrations after 11 months of breastfeeding. The magnitude of relative change predicted by the MDH model aligned well with the middle to upper range of the relative changes in measured serum concentrations from birth to 11 months of age for the 11 children (Figure S1). The mean and 95th percentile of predicted serum concentrations at 11 months of age aligned well with the reported values, differing by <10% (see Supplemental Information).

Transfer of PFOA to infants via breastmilk decreases maternal serum concentrations while increasing infant serum concentrations. Consequently, the concentration of PFOA in breastmilk also decreases over the course of lactation as a portion of the mother’s body burden is transferred to the infant. Based on empirical data, Thomsen and colleagues studied the impact of breastfeeding on PFOA breastmilk concentrations in 10 Norwegian mothers [21]. This study estimated a decrease of 7.7% in breastmilk concentration per month of breastfeeding, which corresponds to a decrease of approximately 47% over 6 months. Empirical data from other sources [8, 22] support Thomsen’s observations, as well as results from MDH’s model that indicates a 40 or 52% decrease over 6 months of breastfeeding using a mean or upper percentile breastmilk intake rate, respectively.

### Use of model to derive HBGV

The model developed by MDH predicts serum concentrations over a person’s lifetime arising directly and/or indirectly (e.g., breastmilk) from water intake. Exposure sources other than ingestion of water are taken into account through the use of an RSC, which allocates a fraction of the RfD to water exposures and the remaining portion to other sources. In the case of PFOA, selection of the appropriate RSC must recognize PFOA’s long elimination half-life. This extended half-life means that past exposures, even ones of short duration, impact contemporary serum concentrations. In addition, the transgenerational transfer from mother to child is also an important factor when selecting the appropriate RSC.

Biomonitoring data from the National Health and Nutrition Examination Survey (NHANES) [23] and the Minnesota East Metro PFC Biomonitoring projects [24], provide high-quality data on PFOA serum concentrations in two relevant populations. Given the long half-life of PFOA, these results can be compared with the ‘reference’ serum concentration of 0.13 mg/L to provide insight into the magnitude of non-water exposures. It should be noted that the ‘reference’ serum concentration is based on population-based parameters and should not be used for clinical assessment or for interpreting serum levels in individuals.

The most recent NHANES biomonitoring data (2013–2014) provides an estimate of serum levels in the US general population of individuals over 12 years of age [23]. NHANES reported a 95th percentile serum concentration of 0.00557 mg/L. Biomonitoring data (2014) for a group of East Metro adult residents who moved into the affected area after a treatment system was installed on the public water supply (i.e., newer residents to the area), show a similar 95th percentile serum value (0.005 mg/L) [24]. Although data for infants are very limited, there are publications regarding serum levels in young children [25–27]. These publications indicate that the 95th percentile values in young children are similar to adult levels. Therefore, available data support the use of 95th percentile values from NHANES and the East Metro newer residents as conservative estimates of non-water ingestion routes of exposure.

MDH uses USEPA’s Exposure Decision Tree methodology [28] to identify an appropriate RSC by subtracting the serum level associated with non-water exposures from the 80% ceiling level ([0.13 mg/L × 0.8] – 0.00557 mg/L = 0.0984 mg/L). This value is approximately 75% of the ‘reference’ serum concentration and represents a residual or maximum serum level that can be apportioned to exposure via ingestion of water. Therefore, an appropriate RSC would be >50% but <80%. Given the limited information regarding non-water exposures in the population of concern (i.e., infants), MDH selected an RSC of 50% for PFOA water ingestion. The resulting serum concentration allocated or ‘allowed’ to result from ingestion of water was 0.065 mg/L (‘reference’ serum concentration of 0.13 mg/L × 0.5). MDH used the model iteratively to identify the water concentration that resulted in a stable or steady-state serum concentration at or below 50% of the ‘reference’ serum concentration (0.065 mg/L) for each of the two RME scenarios shown in Fig. 2.

**Fig. 6:**
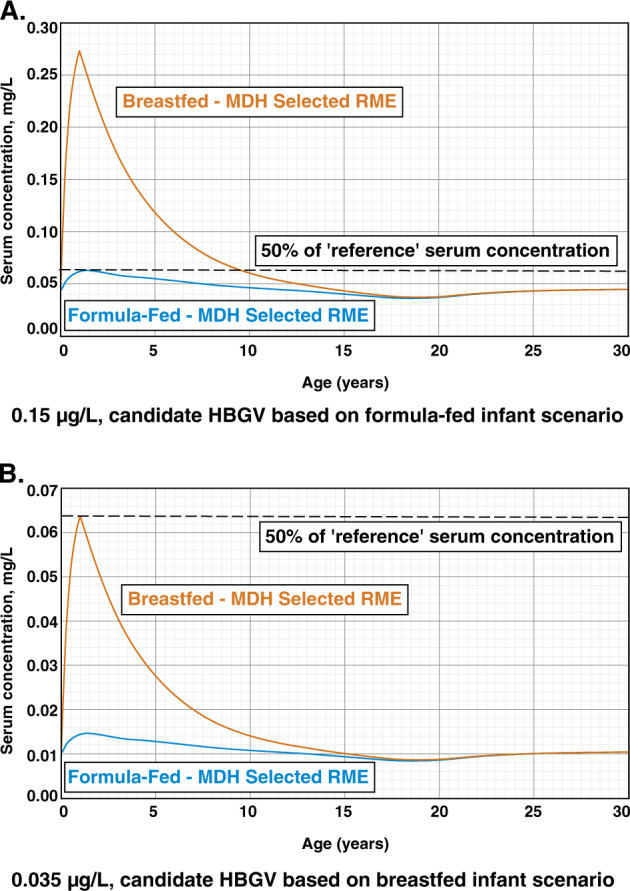
Candidate HBGVs based on PFOA serum concentrations for (**a**) 0.15 µg/L, formula-fed or (**b**) 0.035 µg/L, breastfed scenarios. Note different scale is used for serum concentration in **a** (0–0.3 mg/L) than **b** (0–0.07 mg/L). (Horizontal scale truncated at 30 years to enhance detail)

The water concentration that maintained a PFOA serum concentration at or below 0.065 mg/L throughout life for the formula-fed infant MDH RME scenario was 0.15 µg/L (Fig. 6a). This water concentration, when used in the breastfed infant MDH RME scenario, exceeded the ‘reference’ serum concentration (0.13 mg/L) for >4 years and exceeded 50% of the ‘reference’ serum concentration for >9 years. In order to maintain a PFOA serum concentration at or below 0.065 mg/L, the water concentration had to be lowered to 0.035 µg/L (Fig. 6b). Model simulations using various breastfeeding scenarios that combined different central tendency and upper percentile values for the most sensitive parameters were also assessed (see Table 2) using a water concentration of 0.035 µg/L to ensure that the RME scenario selected by MDH was sufficiently protective.

**Table 2:** Selection of different central (e.g., mean) and upper (e.g., 95th percentile) parameter values for alternative scenario evaluation

Scenario	Intake rate	Breastfeeding duration	Half-life	Transfer rates	Volume of distribution (*V*_d_)	*V*_d_ adjustment factor
MDH RME	Upper	Upper	Central	Central	Central	Central
Alternative 1	Central	Central	Upper	Upper	Central	Central
Alternative 2	Upper	Central	Upper	Central	Central	Central
Alternative 3	Central	Upper	Upper	Central	Central	Central

See Table 1 for actual numerical values used for each parameter

**Fig. 7:**
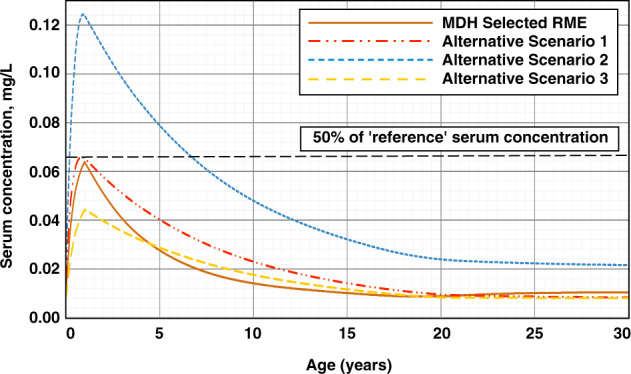
Comparison of MDH selected RME breastfeeding scenario with alternative parameter selection scenarios

The peak serum concentrations for the alternative scenarios ranged from 68% to 96% of the peak serum concentration predicted using the RME scenario selected by MDH (Fig. 7). Based on these results, MDH set final the HBGV for PFOA at 0.035 µg/L, to ensure protection of all segments of the population.

## Discussion

MDH derives HBGVs that are protective of the general population, including sensitive and more highly exposed populations. Addressing higher water intake rates during early life has been integrated into MDH’s current methodology for deriving HBGVs since 2008 [1]. This peer reviewed and promulgated methodology, however, does not address the significant placental and breastmilk transfer and bioaccumulation potential of PFOA. Recent studies have reported compelling evidence that breastfeeding has a significant impact on PFOA serum levels in both nursing infants and their mothers. Empirical data have demonstrated that infant PFOA serum concentrations are higher than those of older individuals exposed to the same contaminated drinking water source [29] and that breastfeeding results in lower PFOA serum concentrations in women and higher concentrations in infants and young children [30]. Despite these observations, PFOA drinking water guidance values derived by other government agencies are typically based on attainment of steady-state serum concentrations from constant exposure over a duration sufficient to achieve steady state (e.g., approximately five half-lives). If this traditional approach were to be used with MDH’s 2017 RfD (0.000018 mg/kg day), RSC (0.5) and a 95th percentile time-weighted average intake rate of 0.064 L/kg per day from birth to 11.5 years of age (half-life of 2.3 years × 5 half-lives to attain steady state), it would result in an HBGV of 0.14 µg/L. This value would be sufficiently protective for formula-fed infants but would be fourfold higher than the water concentration predicted to be protective for breastfed infants. To our knowledge, MDH is the first agency to develop PFOA water guidelines that directly incorporate early life exposure via placental transfer and via breastfeeding.

MDH model parameters have been carefully selected based on the best available science, external peer review comments, and departmental policy. A formal sensitivity analysis of the model was not conducted, however, the limited number of parameters and single-compartment nature of the model lends itself to straightforward decision-making based on the fit-for-purpose concept. Empirical data and modeling studies suggest that half-life, transfer factors, breastfeeding duration, and intake rate are among the most important (impactful) parameters [12]. The current MDH model was evaluated by direct comparison with limited empirical data, which found good agreement. Published pharmacokinetic models also exist and have noted similar dynamics of breastfeeding being a significant source of exposure and early life predicted as having a higher potential for greater serum concentrations of PFOA [11, 12]. Additionally, MDH sought informal input from six external experts regarding the adequacy (e.g., fit-for-purpose) of the model prior to deriving PFOS and PFOA HBGVs in 2017 [16]. Reviewers were not explicitly asked to endorse or approve of the final model. The reviewers’ consensus was that the model was fit-for-purpose, but subject to uncertainties and data gaps that are common to models of this type.

Although PFOA, PFHxS, and PFOS can be excreted through breastmilk, MDH recognizes the important short- and long-term health benefits of breastfeeding for both mother and infant. MDH used an RME scenario to generate HBGVs. An RME scenario depicts a data-driven, realistic, but high-end exposure situation to ensure that even the most heavily exposed individuals within the population will be protected. MDH recommends that women currently breastfeeding, and pregnant women who plan to breastfeed, continue to do so. Exclusive breastfeeding is recommended by doctors and other health professionals for the first 6 months. It is unlikely that potential health concerns from infant PFOA exposure exceed the known benefits of breastfeeding. Application of MDH’s revised HBGVs will ultimately result in lower body burdens and breastmilk concentrations of PFOA so that infants can receive the optimal benefits from breastfeeding.

Among PFAS, PFOA has the largest epidemiological database and, as indicated by serum levels, has been associated with multiple health endpoints, including elevated cholesterol and other serum lipid parameters, as well as liver enzymes, changes in thyroid serum levels and increased incidence of thyroid disease, increased risk of preeclampsia, reduced antibody response, and reduced birth weight [31, 32]. MDH’s ‘reference’ serum concentration is based on laboratory studies where the animals were exposed only to PFOA. These studies found PFOA exposure to cause a variety of health effects, including developmental effects, hepatic toxicity (e.g., effects on lipid metabolism), changes in thyroid hormone levels, and immune system effects. For the human population, where serum is known to contain multiple PFAS, causality has not been established in epidemiological studies. However, consistency of findings across epidemiological studies and concordance with laboratory animal studies raises the level of concern.

PFAS commonly co-occur in drinking water and may have additive health effects. When multiple substances are present, MDH recommends evaluating the potential risk from the combined exposure. Evaluating a mixture of chemicals, based solely on individual HBGVs, may not provide an adequate margin of safety. MDH uses an additive approach, in which chemicals that share a common health endpoint (e.g., liver, developmental) are evaluated together [33]. For each chemical sharing a health endpoint, a ratio of the water concentration of the chemical and the corresponding HBGV is calculated. The ratios are then summed to calculate a health risk index, with any health risk index greater than one receiving further scrutiny.

MDH first released HBGVs for PFOS and PFOA in 2002, PFBA in 2008, and PFBS in 2009. The science regarding PFAS continues to evolve at a rapid pace and MDH has revised their HBGVs several times, most recently in 2017. Currently, six community public water supplies in Minnesota have individual wells above the 2017 revised values. Over 800 homes with private wells have received drinking water well advisories, resulting in either connection to city water or whole-house granular activated carbon filters, which are maintained by the state of Minnesota. Biomonitoring of exposed residents has also been conducted and has demonstrated the effectiveness of treatment systems in reducing or eliminating drinking water exposures to PFAS [34].

Recent estimates conclude that at least 16.5 million people in 36 U.S. states and territories are exposed to PFAS contaminated drinking water, based on USEPA UCMR3 (Unregulated Contaminant Monitoring Rule 3) [32]. It is highly likely that the number of people exposed is higher since this estimate is based on testing of all large (serving > 10,000 people) public water supplies, a limited number of small water supplies, no private drinking water wells, and only six PFAS chemicals. The Minnesota experience with PFAS reinforces a critical need to examine private drinking water wells, while the Organization for Economic Cooperation and Development (OECD) has recently published an updated comprehensive list of over 4700 PFAS-related CAS numbers on the global market [35]. Drinking water surveillance activities are expanding beyond the six PFAS chemicals included in USEPA UCMR3 (PFBS, PFHxS, PFOS, PFOA, perfluorononanoic acid, and perfluoroheptanoic acid), and analytical detection limits continue to improve. Although the national spotlight has only recently been cast upon PFAS in drinking water, based on Minnesota’s decade and a half of experience, concerns regarding these chemicals as groundwater contaminants are likely to persist and grow in prominence.

## Supplementary information

Supplemental Information
