# Correction: A transgenerational toxicokinetic model and its use in derivation of Minnesota PFOA water guidance

**DOI:** 10.1038/s41370-019-0148-z

**Published:** 2019-06-11

**Authors:** Helen M. Goeden, Christopher W. Greene, James A. Jacobus

**Affiliations:** Minnesota Department of Health, 625 Robert St. N, P.O. Box 64975, St. Paul, MN 55164-0975 USA

**Correction to:** **Journal of Exposure Science & Environmental Epidemiology**

10.1038/s41370-018-0110-5

published online 10 January 2019

In the original Article, References 34–36 listed incorrect URLs. The correct URLs are:

34. MDH (Minnesota Department of Health). East Metro PFC3 biomonitoring project: December 2015. Report to the Community. 2015b. https://www.health.state.mn.us/communities/environment/biomonitoring/docs/pfc2015communityreport.pdf

35. OECD (Organization for Economic Co-operation and Development). Toward a new comprehensive global database of per and polyfluoroalkyl substances (PFASs): summary report on updating the OECD 2007 list of per- and polyfluoroalkyl substances (PFASs). 2018. http://www.oecd.org/officialdocuments/publicdisplaydocumentpdf/?cote=ENV-JM-MONO(2018)7&doclanguage=en

36. USEPA (US Environmental Protection Agency). National Center for Environmental Assessment. Exposure Factors Handbook. Edition. 2011. https://19january2017snapshot.epa.gov/expobox/exposure-factors-handbook-2011-edition_.html

