# Correction to: A transgenerational toxicokinetic model and its use in derivation of Minnesota PFOA water guidance

**DOI:** 10.1038/s41370-019-0191-9

**Published:** 2019-11-25

**Authors:** Helen M. Goeden, Christopher W. Greene, James A. Jacobus

**Affiliations:** Minnesota Department of Health, 625 Robert St. N, P.O. Box 64975, St. Paul, MN 55164-0975 USA

**Correction to: Journal of Exposure Science & Environmental Epidemiology**

10.1038/s41370-018-0110-5

This paper has been corrected because the model runs used to create the serum curves for the Alternative Scenarios in Fig. 7 contained errors, resulting in an underestimation of serum concentrations. The errors did not affect the model results for the MDH selected RME scenario, which was used to develop Minnesota’s water guidance values. The corrected Fig. 7 is presented below along with revised text for the last three sentences of the Results section.

**Figure 1:**
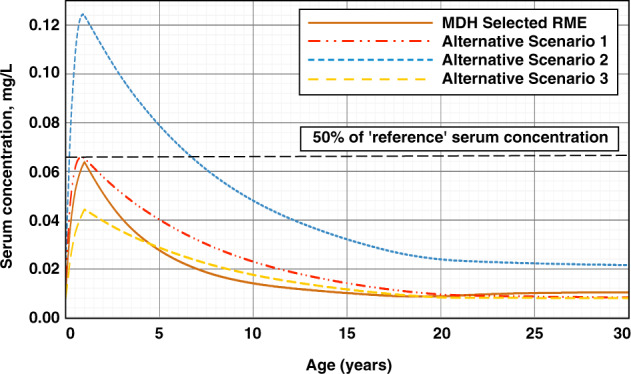
Fig. 7

Model simulations of breastfeeding scenarios that combined different central tendency and upper percentile values for various model parameters were also assessed (see Table 2) using MDH’s HBGV of 0.035 µg/L.

The peak serum concentrations for the alternative scenarios ranged from 69 to 195% of the peak serum concentration predicted using the RME scenario selected by MDH (Fig. 7). While these alternative scenarios produce similar or somewhat higher peak serum concentrations, these estimates were used for comparison purposes only to better understand the most sensitive model parameters.

The updates to this Article take effect in both the HTML and PDF versions.

