# Supplementary material for: A transgenerational toxicokinetic model and its use in derivation of Minnesota PFOA water guidance
Source: J Expo Sci Environ Epidemiol. 2019 Jan 10;29(2):183–95. doi: 10.1038/s41370-018-0110-5 (PMC6760606; doi:10.1038/s41370-018-0110-5)
Supplement: Supplementary file 1 — Supplemental Information [file 41370_2018_110_MOESM1_ESM.docx]

**Supplemental Information:**

**Manuscript Title: A Transgenerational Toxicokinetic Model and Its Use in Derivation of Minnesota PFOA Water Guidance**

**Authors: Helen M. Goeden^1,2^ PhD, Christopher W. Greene^1^ MS, and James A. Jacobus^1^ PhD**

**^1^**Minnesota Department of Health, 625 Robert St. N, P.O. Box 64975, St. Paul, MN 55164-0975

**^2^**Corresponding author: Helen Goeden (email: [helen.goeden@state.mn.us](mailto:helen.goeden@state.mn.us); phone: 651-201-4904; FAX 651-201-4727

**Description**

Table S1. Summary of PFOA half-life study information.

Table S2. Summary of PFOA placental transfer study information.

Table S3. Summary of PFOA breastmilk transfer study information.

Figure S1 and S2. Comparison of MDH Model Results with Mogensen et al 2015

References

**Table S1. Summary of PFOA half-life study information.** Key study selected in **bold**.

| **Study** | **Population size and Location** | **Age**  **(yrs)** | **Gender** | **Initial Serum Level (ng/mL)** | **Half-life Estimate (yrs)** | **Description** |
| --- | --- | --- | --- | --- | --- | --- |
| Li et al 2018 (1) | 106  (20 males age 15–50; 30 females age 15–50)  Sweden | 4–83 | 50 male (M)/  56 female (F) | Mean 21.1  (range 2.4–92) | Mean  2.7 (all ages)  2.8 (M age 15–50)  2.4 (F age 15–50)  (5^th^–95^th^ percentile range 1.8–5.1) | Community exposed to contaminated drinking water. Six to seven samples per person taken over 28 month period. Authors noted that interindividual variation was substantial, with a 3-fold difference between 5^th^ and 95^th^ percentile, plus a few outliers with extremely long half-lives. |
| Worley et al 2017 (2) | 45 (2016)  Alabama, USA | Mean  62.6 (2016) | 22 M/23 F (2016) | Not reported for subgroup of 45 at initiation (2010)  Entire cohort (n=153) Geometric mean 16.3 (95^th^ percentile 61.1) | Mean 3.9 | Community exposed via agricultural use of contaminated sewage sludge, which resulted in environmental contamination. Two samples per person taken, one in 2010 and one in 2016. Geometric mean serum levels for the subgroup of 45 at the end of the study period in 2016 was 11.7 ng/mL (95^th^ percentile 39.1 ng/mL) |
| Gomis et al 2017 (3) | Australia and USA nationwide biomonitoring data, specifics  not reported | Not reported | Not reported | Not reported | Mean  United States  2.4 M  2.1 F  Australia  2 M  1.8 F | Cross-sectional biomonitoring (e.g., NHANES for United States estimate) and population based pharmacokinetic modeling based estimates. |
| Fu et al 2016 (4) | 302  China | Mean  41  (19-65 range) | 213 M/89 F | Median 427 | Geometric mean  4.1  (clearance-based)  1.7  (annual decline based) | Occupational workers. Estimated for individuals based on daily clearance or annual decline (2008-2012) |
| MDH 2015a (5) and Nelson 2016 (6) | 149  East Metro area,  Minnesota,  USA | mean 53 | Not reported | Geometric mean 11.2  (2010) | 3.4  based on individual results  3.2  based on geometric means | Community exposed to contaminated water. Three samples per person taken in 2008, 2010 and 2014. Elimination rates (e.g., half-life) were estimated using 2010 and 2014 data points. Authors cautioned that not accounting for all exposure sources could influence elimination rate estimates. |
| Seals et al 2011 (7) | 602  Little Hocking,  Ohio, USA  971  Lubeck,  West Virginia, USA | Not reported | Not reported | Median  241  (Little Hocking)  69.4  (Lubeck) | Mean  2.9  (range 2.5–3.0)  (Little Hocking)  8.5  (range 5.9–10.3)  (Lubeck) | Cross-sectional study in community exposed to contaminated water. |
| **Bartell et al 2010 (8)** | **200**  **(172 public system, 28 bottled water)**  **Ohio and**  **West Virginia,**  **USA** | **mean 54.5**  **(range 18–89+)** | **approx. equal male & female** | **Mean**  **180** | **Mean  2.3**  **(95% of individuals expected to be between 1.5 and 4.6)** | **Community exposed to contaminated drinking water. Eight samples per person taken over a 1-year period, beginning in June 2007. Authors note that subject specific half-lives varied widely.** |
| Brede et al 2010 (9) | 65  (20 children, 22 mothers, 23 men)  Arnsberg, Germany | Children:  mean 7.9 (range  7.4–8.3)  Mothers:  mean 38.1 (range  27–49)  Men:  mean 55.2  (range  32–71) | Children  9 M/11 F  Adults  23 M/22 F | 50^th^ percentile  Children  22.4  Mothers  25.1  Men  32.8 | Geometric mean 3.26  (range 1.0–14.7) | Community exposed to contaminated water. Two samples per person over 2 years. |
| Olsen et al 2007 (10) | 26  Alabama, USA: 23  Minnesota, USA: 3 | mean 61 at study initiation  (range  55–75) | 24 M  2 F | Mean 691  (range  72–5,100) | Median  3.4  (range 1.5–9.1)  Arithmetic mean 3.8  Geometric mean 3.5  (calculated 5^th^–95^th^ percentile range 1.7–7.0) | Retired workers. Seven to eight samples per person taken over a 5-year period (1999–2004). |

**Table S2. Summary of PFOA placental transfer study information.**

| **Study** | **Study Description** | **Cord:Maternal Serum Concentration** | | | |
| --- | --- | --- | --- | --- | --- |
|  |  | **Mean Ratio** | **Median Ratio** | **Maximum Ratio** | **95^th^ Percentile Ratio** |
| *Transfer Rates Based on Paired Ratio Data Reported by Authors* | | | | | |
| Manzano-Salgado et al 2015 (11) | Measured PFBS, PFHxS, PFOS, PFOA and PFNA in maternal and cord serum from 66 mother-child pairs from a Spanish birth cohort. The geometric mean for paired maternal:cord ratios was reported by authors in Table 2. Ratio presented here was inverted here to represent Cord:Maternal Ratio. NOTE: maternal samples may have been taken at week 12 of pregnancy and not at or near delivery time. | 0.75 |  |  |  |
| Cariou et al 2015 (12) | Maternal serum, cord serum and breastmilk were obtained from 102 female volunteers hospitalized between June 2010 and Jan 2013 for planned caesarean delivery. Maternal blood and cord samples collected at time of delivery. Final number of samples were: 100 for maternal serum and 106 cord serum (including 6 twin pairs). Study was not designed as an epidemioligical study but as an exploratory study to explore exposure trends to guide further studies. Table 3 reports mean, standard deviation (SD), minimum and maximum cord:maternal serum ratios. Ninety-fifth percentile estimated (mean + 2SD assuming normal distribution) from data provided in Table 3. | 0.78 |  | 1.52 | 1.11 |
| Lee et al 2013 (13) | Maternal serum and umbilical cord blood concentrations were collected from 59 pairs of maternal and umbilical cord sera in South Korea from March through August 2011. Mean, SD, minimum, maximum, as well as 25^th^, 50^th^, and 75^th^ percentile paired maternal:cord ratios were reported by authors in Table 4. Ninety-fifth percentile was estimated from data provided (mean + 2SD). | 0.84 |  | 2.16 | 1.44 |
| Porpora et al 2013 (14) | Described as a preliminary study. PFOS and PFOA levels were determined in serum samples of 38 pregnant women living in Rome, Italy. Maternal levels were determined at time of hospitalization or the next hours after deliver. Cord blood of their newborns was taken at delivery. Study was carried out in 2008-2009. | 0.87 |  | 1.77 |  |
| Beesoon et al 2011 (15) | Subset of Chemicals, Health, and Pregnancy (CHirP) cohort recruited in 2007 in Vancouver, BC, Canada. (N=20). Participants provided blood sample at 15 weeks of gestation and samples of cord blood at deliver. Maternal levels at 15 weeks were adjusted to time of delivery. Unadjusted mean and maximum ratios were 0.61 and 0.96, respectively. Adjusted mean ratio was also reported by authors. | 0.71  adjusted |  |  |  |
| Kim et al 2011a (16) | Pregnant women were recruited from three hospitals located in Seoul, Cheongju, and Gumi, South Korea between August 2008 and March 2009. Blood samples drawn from 44 pregnant women, mostly during the 3^rd^ trimester. Cord blood (N=43) drawn at delivery, 35 of which were collected from matching mother-infant pairs. Breastmilk (N=35) collected during mother's check-up ~1 month after delivery. Matching maternal blood-cord blood-milk samples obtained from 26 mother-infant pairs. Paired data was not in publication. Authors reported mean paired ratio. | 1.02 |  |  |  |
| Kim et al 2011b (17) | Twenty volunteers from Seoul donated maternal serum, umbilical cord serum, and breast milk in 2007. Maternal serum was obtained one day before delivery and umbilical cord serum was collected immediately after delivery. (N=20 for maternal serum & umbilical cord). Ratio reported by authors in text. | 0.69 |  |  |  |
| Liu et al 2011 (18) | Matched maternal serum, cord serum and breast milk samples were collected from 50 pairs of women and their newborns from Jiang Su province of China in June and July 2009. Cord blood samples collected immediately after delivery and maternal blood samples were collected within first week after delivery. Summary statistics are reported in Table 2. Table 3 reported mean and median cord:maternal serum ratios. | 0.91 | 0.89 | 1.63 |  |
| Needham et al 2011 (19) | Paired mother-child samples were collected to measure the concentration of 87 environmental chemicals, including five PFAS compounds. Mothers (with normal parturition) from the Faroe Islands were chosen because of the anticipated wide range of exposure due to the traditional diet, which includes pilot whale. Samples were obtained in the year 2000. PFAS compounds were analyzed on 12 sets of maternal serum, cord serum, and milk. Ratios reported by authors. | 0.72 |  |  |  |
| Fromme et al 2010 (20) | Maternal blood, cord blood, infant blood, & breast milk were collected between December 2007 and October 2009 in Munich, Germany. Study population consisted of randomly selected females participating in a birthing class. 38 Maternal blood samples collected @ 34-37^th^ week of pregnancy, at delivery, and 6 months after birth. 33 Cord blood samples collected @ delivery. Infant blood collected @ approximately 6 and 19 months of age. Cord:Maternal ratio reported by authors in Table 2. | 0.70 |  |  |  |
| Midasch et al 2007 (21) | Plasma levels of 11 women a few hours prior to childbirth and corresponding cord plasma levels of 11 neonates were collected between March and April 2003 in Germany. Authors reported that PFOA crosses the placental barrier unhindered. Individual pair ratios as well as mean and median ratios were reported in Table 1. Ninety-fifth percentile was estimated from data provided (mean + 2SD). | 1.26 | 1.22 | 1.95 | 1.69 |
| Fei et al 2007 (22) | Danish National Cohort study, which included pregnant women participants from March 1996 to Nov 2002. Fifty mother:infant pairs were randomly selected to evaluate correlation between maternal and offspring levels. Two maternal blood samples were taken, once in the first trimester and once in the second trimester. Second trimester levels used in comparison. Cord blood was obtained shortly after birth. Mean second trimester maternal:cord concentration ratio of 1.46 was reported in text. This was inverted to generate a cord:maternal ratio of 0.68. | 0.68 |  |  |  |
| Summary statistics based on reported paired ratio statistics: | | | | | |
| N  Minimum  Maximum  Mean  Geometric Mean  95^th^ Percentile | | 12 | 2 | 5 | 3 |
|  |  | 0.68 | 0.89 | 1.52 | 1.11 |
|  |  | 1.26 | 1.22 | 2.16 | 1.69 |
|  |  | 0.83 |  | 1.81 | 1.41 |
|  |  | 0.81 |  | 1.79 | 1.39 |
|  |  | 1.13 |  | 2.12 | 1.67 |
| Transfer Rates Calculated from Summary Statistics | | | | | |
| Chen et al 2017 (23) | Paired maternal serum and umbilical cord serum were collected from 32 pregnant women in Wuhan, China between November 2015 and March 2016. Calculated from summary statistics presented in Table S4. | 0.79 |  |  |  |
| Zhang et al 2013 (25) | Eleven PFAS were analyzed in matched samples, including maternal blood, cord blood, placenta and amniotic fluid from 32 women recruited at a hospital in Tianjin, China in 2010. Calculated from Table 1. | 0.58 |  |  |  |
| Gutzkow et al 2012 (26) | Participants were part of the MoBa cohort in Norway. For this evaluation, 213 paired samples of maternal and cord blood were collected at the hospital immediately after birth. Ratios calculated from summary data provided in Table 1. | 0.82 |  |  |  |
| Kim et al 2011b (17) | Twenty volunteers from Seoul donated maternal serum and umbilical cord serum in 2007. Maternal serum was obtained one day before delivery and umbilical cord serum was collected immediately after delivery (N=20 for maternal serum and umbilical cord). Mean ratio reported in text but 95^th^ percentile ratio calculated from summary data provided. |  |  |  | 0.84 |
| Monroy et al 2008 (27) | Study was nested within a larger ongoing study (Family Study) in Ontario, Canada. Maternal samples taken during the second trimester and again at delivery. Cord samples taken at delivery. (N=101 mothers, N=105 newborns). Mean calculated from Table 3. | 0.87 |  |  |  |

**Table S3. Summary of PFOA breastmilk transfer study information.**

| **Study** | **Study Description** | **Breastmilk:Maternal Serum Concentration** | | |
| --- | --- | --- | --- | --- |
|  |  | **Mean Ratio** | **Median Ratio** | **Maximum Ratio** |
| *Transfer Rates Based on Paired Ratio Data Reported by Authors* | | | | |
| Cariou et al 2015 (12) | Maternal serum, cord serum and breast milk were obtained from 102 female volunteers hospitalized between June 2010 and January 2013 for planned caesarean delivery. Maternal blood and cord samples collected at time of delivery. Breast milk collected between 4th and 5th day after delivery. Final number of samples 100 for maternal serum, 106 cord serum (including 6 twin pairs), and 61 breast milk. Study was not designed as an epidemiological study but as an exploratory study. | 0.038 |  |  |
| Liu et al 2011 (18) | Matched maternal serum, cord serum and breast milk samples were collected from 50 pairs of women and their newborns from Jiang Su province of China between June and July 2009. Cord blood samples collected immediately after delivery, maternal blood samples collected within first week after delivery and breast milk samples within one week of delivery. Table 3 reported mean and median ratios. | 0.11 | 0.09 |  |
| Kim et al 2011a (16) | Pregnant women were recruited at three hospitals located in Seoul, Cheongju, and Gumi, South Korea between August 2008 and March 2009. Blood samples drawn from 44 pregnant women, mostly during the third trimester. Cord blood (N=43) drawn at delivery, 35 of which were collected from matching mother-infant pairs. Breastmilk (N=35) collected during mother's check-up approximately 1 month after delivery. Matching maternal blood-cord blood and breastmilk samples were obtained for 26 mother-infant pairs. Median ratio reported in Table 4. |  | 0.040 |  |
| Kim et al 2011b (17) | Twenty volunteers from Seoul donated maternal serum, umbilical cord serum, and breast milk in 2007. Maternal serum was obtained 1 day before delivery and umbilical cord serum was collected immediately after delivery. Breast milk was collected 3-10 days after delivery. (N=20 for maternal serum & umbilical cord; N=17 for breastmilk). Mean ratio reported by authors in text. | 0.025 |  |  |
| Haug et al 2011 (28) | Forty-one female volunteers from Oslo, Norway area donated a serum sample and approximately half (N=19) provided a sample of breast milk. Serum samples were collect between August 2007 and May 2008. Breast milk samples were collected between August 2007 and September 2008. Serum and breastmilk levels were reported in Supplemental Table 6. Authors reported average breastmilk concentrations were 3.8% of the corresponding serum concentrations. | 0.038 |  |  |
| Karrman et al 2007 (29) | Matched individual milk and serum samples from 12 primiparous Swedish women in 2004 were measured and evaluated. Ratios are reported in Table 3. | 0.12 |  |  |
| Summary statistics based on reported paired ratio statistics: | | | | |
| N  Minimum  Maximum  Mean  Geometric Mean  95^th^ Percentile | | 5 | 2 | 0 |
|  |  | 0.025 | 0.040 | NA |
|  |  | 0.120 | 0.090 | NA |
|  |  | 0.066 |  | NA |
|  |  | 0.054 |  | NA |
|  |  | 0.118 |  | NA |

**Supplemental Figure S1 and S2. Comparison of MDH Model Results with Mogensen et al 2015^(30)^.**

Two comparisons were conducted: 1) magnitude of relative change in infant serum concentrations from birth to 11 months of age; and 2) modeling of individual infant serum concentrations after 11 months of breastfeeding. MDH used WebPlotDigitizer to estimate serum concentrations for PFOA at birth and at 11 months of age from curves for each of the 11 children who were at least partially breastfed (as presented in Mogensen’s Figure 1^(30)^). These data are not ideal for comparison for three reasons:

1. infant serum concentrations at birth were not measured but estimated from the maternal concentrations by the authors using a single factor value of 0.34;
2. children drank only breastmilk for a median duration of 4.5 months, followed by partial breastfeeding with supplementary baby food for a median duration of 4 months, and data are not available to ascertain these durations for each individual; and
3. infant serum samples were not taken until 11 months of age.

For the first analysis, the magnitude of the relative change predicted by the MDH model was compared to those in Mogensen Figure 1^(30)^. The magnitude of relative change predicted by the MDH model (for breastfed infants) fell within the middle to upper range of the relative magnitude changes in serum concentrations from birth to 11 months of age for the 11 children (Figure S1).

**Figure S1. Relative increase in infant PFOA serum concentration at 11 months of age normalized to concentration at birth. MDH model results for breastfed infant vs estimated individual data points from Mogensen (Figure 1^(30)^). Upper and mean breastmilk intake rates derived from USEPA^(31)^ (See Table 1).**


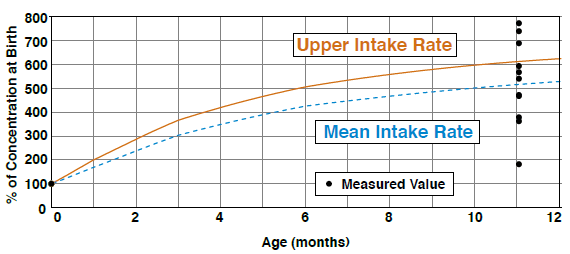


For the individual infant comparison, the corresponding maternal serum concentration, breastmilk concentration, and ongoing maternal exposure were calculated using MDH RME model parameter values as described for the comparison with Fromme et al. The mean and 95^th^ percentile of the reported infant serum concentrations at 11 months of age were 9.7 and 15.1 µg/L, respectively. The mean and 95^th^ percentile of the model predicted values at 11 months of age were 9.8 and 13.8 µg/L, respectively. The comparison of individual modeled to measured infant serum concentration resulted in a R^2^ of 0.4397 (Figure S2). The objective of the model is to predict reasonable upper percentile population-based serum concentrations under the selected RME scenario. This scenario includes breastfeeding for 11 months. Despite the gradual and unknown transition away from breastfeeding prior to six months in the empirical data, as well as the long interval between cessation of breastfeeding and sampling of serum, our model results compare reasonably well with the Mogensen data.

**Figure S2. Predicted individual infant PFOA serum concentrations at 11 months of age versus measured levels estimated from Mogensen (Figure 1^(30)^).**

**
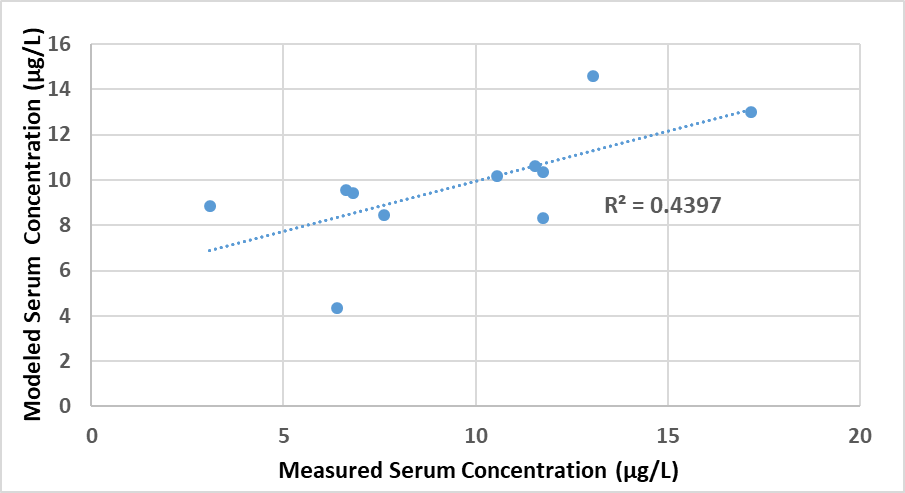
**

**References**

1. Li Y, T Fletcher, D Mucs, K Scott, CH Lindh, P Tallving, K Jakobsson. Half-lives of PFOS, PFHxS and PFOA after end of exposure to contaminated drinking water. Occupational and Environmental Medicine. 2018;75:46-51.

2. Worley R, SM Moore, BC Tierney, X Ye, AM Calafat, S Campbell, MB Woudneh, J Fisher. Per- and polyfluoroalkyl substances in human serum and urine samples from a residentially exposed community. Environment International. 2017;106:135-43.

3. Gomis M, R Vestergren, M MacLeod, JF Mueller, IT Cousins. Historical human exposure to perfluoroalkyl acids in the United States and Australia reconsturcted from biomonitoring data using population-based pharmacokinetic modelling. Environment International. 2017;108:92-102.

4. Fu J, Y Gao, T Wang, Y Liang, G Qu, B Yuan, Y Wang, A Zhang, G Jiang. Occurrence, temporal trends, and half-lives of perfluoroalkyl acids (PFAAs) in occupational workers in China. Scientific Reports. 2016;6:38039:DOI: 10.1038/srep38039.

5. MDH. Minnesota Department of Health. Environmental Health & Biomonitoring Advisory Panel June 9, 2015 Meeting Background Materials. 2015a.

6. Nelson J. Personal Communication regarding MDH MN (East Metro) PFC biomonitoring project data based on June 9, 2015 Meeting Agenda and Materials for the Advisory Panel to the Environmental Health Tracking and Biomonitoring Program. <http://www.health.state.mn.us/divs/hpcd/tracking/panel/2015Junematerials.pdf>. 2016.

7. Seals R, SM Bartell, K Steenland. Accumulation and Clearance of Perfluorooctanoic Acid (PFOA) in Current and Former Residents of an Exposed Community. Environmental Health Perspectives. 2011;119:119-24.

8. Bartell S, AM Calafat, C Lyu, K Kato, PB Ryan, K Steenland. Rate of Decline in Serum PFOA Concentrations after Granular Activated Carbon Filtration at Two Public Water Systems in Ohio and West Virginia. Environmental Health Perspectives. 2010;118:222-8.

9. Brede E, M Wilhelm, T Goen, J Muller, K Rauchfuss, M Kraft, J Holzer. Two-year follow-up biomonitoring pilot study of residents' and controls' PFC plasma levels after PFOA reduction in public water system in Arnsberg, Germany. International Journal of Hygiene and Environmental Health. 2010;213:217-23.

10. Olsen G, JM Burris, DJ Ehresman, JW Froehlich, AM Seacat, JL Butenhoff, LR Zobel,. Half-life of Serum Elimination of Perfluorooctanesulfonate, Perfluorohexanesulfonate, and Perfluorooctanoate in Retired Fluorochemical Production Workers. Environmental Health Perspectives. 2007;115:1298-305.

11. Manzano-Salgado C, M Casas, MJ Lopez-Espinosa, F Ballester, M Basterrechea, JO Grimalt, AM Jimenez, T Kraus, T Schettgen, J Sunyer, M Vrijheid. Transfer of perfluoroalkyl substances from mother to fetus in a Spanish birth cohort. Environmental Research. 2015;142:471-8.

12. Cariou R, B Veyrand, A Yamada, A Berrebi, D Zalko, S Durand, C Pollono, P Marchand, J-C Leblanc, J-P Antignac, B Le Bizec. . Perfluoroalkyl acid (PFAA) levels and profiles in breast milk, maternal and chord serum of French women and their newborns. Environment International. 2015;84:71-81.

13. Lee Y, M-K, Kim, J Bae, J-H Yang. Concentrations of perfluoroalkyl compounds in maternal and umbilical cord sera and birth outcomes in Korea. Chemosphere. 2013;90:1603-9.

14. Porpora M, R Lucchini, A Abballe, AM Ingelido, S Valentini, E Fuggetta, V Cardi, A Ticino, V Marra, AR Fulgenzi, E De Felip. Placental Transfer of Persistent Organic Pollutants: A Preliminary Study on Mother-Newborn Pairs. International Journal of Environmental Research and Public Health. 2013;10:699-711.

15. Beesoon S, GM Webster, M Shoeib, T Harner, JP Benskin, JW Martin. Isomer Profiles of Perfluorochemicals in Matched Maternal, Cord, and House Dust Samples: Manufacturing Sources and Transplacental Transfer. Environmental Health Perspectives. 2011;119:1659-64.

16. Kim S, K Choi, K Ji, J Seo, Y Kho, J Park, S Kim, S Park, I Hwang, J Jeon, H Yang, JP Giesy. Trans-Placental Transfer of Thirteen Perfluorinated Compounds and Relations with Fetal Thyroid Hormones. Environmental Science & Technology. 2011a;45:7465-72.

17. Kim S-K, KT Lee, CS Kang, L Tao, K Kannan, KR Kim, CK Kim, JS Lee, PS Park, YW Yoo, JY Ha, YS Shin, JH Lee. Distribution of perfluorochemicals between sera and milk from the same mothers and implications for prenatal and postnatal exposures. Environmental Pollution. 2011b;159:169-74.

18. Liu J, J Li, Y Liu, HM Chan, Y Zhao, Z Cai, Y Wu. Comparison on gestation and lactation exposure of perfluorinated compounds for newborns. Environment International. 2011 37:1206-12.

19. Needham L, P Grandjean, B Heinzow, PJ Jorgensen, F Nielsen, DG Patterson Jr, A Sjodin, WE Turner, P Weihe. Partition of Environmental Chemicals between Maternal and Fetal Blood and Tissues. Environmental Science & Technology. 2011;45:1121-6.

20. Fromme H, C Mosch, M Morovitz, I Alba-Alejandre, S Boehmer, M Kiranoglu, F Faber, I Hannibal, O Genzel-Boroviczeny, B Koletzko, W Volkel. Pre- and Postnatal Exposure to Perfluorinated Compounds (PFCs). Environmental Science & Technology. 2010;44:7123-9.

21. Midasch O, H Drexler, N Hart, MW Beckmann, J Angerer. Transplacental exposure of neonates to perfluorooctanesulfonate and perfluorooctanoate: a pilot study. International Archives of Occupational and Environmental Health. 2007;80:643-8.

22. Fei C, JK McLaughlin, RE Tarone, J Olsen. Perfluorinated Chemicals and Fetal Growth: A Study within the Danish National Birth Cohort. Environmental Health Perspectives. 2007;115(11):1677-82.

23. Chen F, S Yin, BC Kelly, W Liu. Isomer-Specific Transplacental Transfer of Perfluoroalkyl Acids: Results from a Survey of Paired Maternal, Cord Sera, and Placentas. Environmental Science & Technology. 2017;51:5756-63.

24. Kato K, L-Y Wong, A Chen, C Dunbar, GM Webster, BP Lanphear, AM Calafat. Changes in Serum Concentrations of Maternal Poly- and Perfluoroalkyl Substances over the Course of Pregnancy and Predictors of Exposure in a Multiethnic Cohort of Cincinnati, Ohio Pregnant Women during 2003-2006. Environmental Science & Technology. 2014;48:9600-8.

25. Zhang T, H Sun, Y Lin, X Qin, Y Zhang, X Geng, K Kannan. Distribution of Poly- and Perfluoroalkyl Substances in Matched Samples from Pregnant Women and Carbon Chain Length Related Maternal Transfer. Environmental Science & Technology. 2013;47:7974-81.

26. Gutzkow K, LS Haug, C Thomsen, A Sabaredzovic, G Becher, G Brunborg. Placental transfer of perfluorinated compounds is selective - A Norwegian Mother and Child sub-cohort study. International Journal of Hygiene and Environmental Health. 2012;215:216-9.

27. Monroy R, K Morrison, K Teo, S Atkinson, C Kubwabo, B Stewart, WG Foster,. Serum levels of perfluoroalkyl compounds in human maternal and umbilical cord blood samples. Environmental Research. 2008;108:56-62.

28. Haug L, S Huber, G Becher, C Thomsen,. Characterisation of human exposure pathways to perfluorinated compounds - Comparing exposure estimates with biomarkers of exposure. Environment International. 2011;37:687-93.

29. Karrman A, I Ericson, B van Bavel, PO Darnerud, M Aune, A Glynn, S Lignell, G Lindstrom. Exposure of Perfluorinated Chemicals through Lactation: Levels of Matched Human Milk and Serum and a Temporal Trend, 1996-2004, in Sweden. Environmental Health Perspectives. 2007;115:226-30.

30. Mogensen U, P Grandjean, F Nielsen, P Weihe, E Budtz-Jorgensen,. Breastfeeding as an Exposure Pathway for Perfluorinated Alkylates. Environmental Science & Technology. 2015;49:10466-73

31. USEPA (US Environmental Protection Agency). National Center for Environmental Assessment. Exposure Factors Handbook. 2011 Edition. 2011.
